# Sarcopenia

**DOI:** 10.1111/1753-0407.70025

**Published:** 2024-10-29

**Authors:** Zachary Bloomgarden

**Affiliations:** ^1^ Department of Medicine Division of Endocrinology, Diabetes and Bone Disease, Icahn School of Medicine at Mount Sinai New York New York USA

The term “sarcopenia” literally means “deficiency of flesh,” and is used to refer to lack of skeletal muscle. Numerous similar concepts in medicine describe the progressive loss, deficiency, atrophy, or wastage of muscle characteristic of many systemic illnesses and of aging itself. Depending on the definition used, sarcopenia affects large subsets of the population, in association with physical inactivity, cigarette smoking, and malnutrition but also paradoxically with obesity. Sarcopenia is seen with diabetes, pulmonary disease, heart disease, malignancy, and with psychiatric and neurologic illnesses including depression/anorexia and Alzheimer's and Parkinson's diseases.^1^ In a study carried out nearly four decades ago, total appendicular skeletal muscle mass was found to decrease in a linear fashion with age both among men and women, regardless of race or ethnic group, showing positive correlation with body weight.^2^ Sarcopenia can be assessed clinically with measures of strength such as the simple self‐report of limitation of walking, which increases in prevalence with increasing age, to a greater extent in low‐ than in high‐income countries, and which correlates strongly with all‐cause mortality even after adjustment for age, sex, education, marital status, rural residence, and country income level, and additionally for hypertension, diabetes, coronary artery disease, stroke, body mass index (BMI), smoking, physical activity, and depression.^3^ In a study analyzing mortality at >12 year follow‐up, those in the highest quintile of the fat‐to‐muscle mass ratio estimated using bioelectrical impedance among 337 951 UK Biobank participants had increased total and cardiovascular disease (CVD) mortality, both among men and women.^4^

Clinical conditions associated with sarcopenia overlap with features of frailty such as slowing, falls, fatigue, and weight loss, which may represent disease prodromes,^5^ with a continuum from robustness, with stressors leading to temporary decline in functional capacity, to pre‐frailty, with only incomplete recovery from stressors, to actual frailty with failure to recover from stressors eventuating in states of dependence and disability.^6^ The biology of frailty involves a number of factors associated with sarcopenia, including states of dysregulated nutrient sensing, such as abnormalities of mammalian target of rapamycin (mTOR) complex 1, AMP‐activated protein kinase (AMPK), and the nutrient scarcity sensors sirtuins 1 and 3, and hormonal changes associated with aging including decreased levels of the anabolic hormones dehydroepiandrosterone sulfate, testosterone, growth hormone, and insulin‐like growth factor 1, and increased levels of catabolic hormones, particularly cortisol.^7^ Insulin can best be seen in this context as an anabolic hormone involved in nutrient storage, but rather than being deficient, it is typically present in excess in association with insulin resistance leading to reduction in its anabolic functional activity.^8^

Sarcopenia is associated with chronic inflammation, mediated by cellular senescence and mitochondrial dysfunction, inhibiting growth factor expression, and causing DNA damage and oxidative stress increasing catabolism.^7^ Inflammation is further potentiated by induction of apoptotic pathways, by insulin resistance and visceral obesity, leading to elevated circulating levels of proinflammatory lipid‐derived molecules, and by loss of antioxidant defenses including superoxide dismutase; GSH; vitamins E, C, and A; uric acid; and thioredoxin.^9^ In a Mendelian randomization analysis of cytokines related to sarcopenia, associations were found for IL‐7, which acutely upregulates lipolysis and fat oxidation and is associated chronically with decreased muscle strength, for monocyte chemotactic protein 3, and for regulated on activation, normal T cell expressed and secreted (RANTES), which contributes to aggregation of inflammatory cells and to persistence of the inflammatory response; in addition, several cytokines were identified which appear to play protective roles but which are deficient in states of sarcopenia, including hepatocyte growth factor (HGF), which increases skeletal muscle regeneration by regulating mobilization and modification of bone marrow stem cells; interferon gamma–induced protein (IP)‐10, involved in muscle regeneration; and macrophage colony‐stimulating factor (M‐CSF), which appears to increase muscle contractile strength.^10^ Bone‐derived cytokines (osteokines) appear to play roles similar to those of skeletal muscle–derived myokines, with mesenchymal precursor cells referred to as fibro‐adipogenic progenitors (FAPs) generating adipocytes, fibroblasts, osteoblasts, and myocytes.^11^ Abnormal FAP differentiation leads to decreased muscle regeneration in association with a dysregulated immune response, leading to persistent low‐grade inflammation and excessive fat infiltration and fibrosis.^11^ Diabetes is associated with defects in muscle regeneration with reduction in muscle stem cells and angiogenesis and increase in FAP fibrosis,^12^ the balance shifting to adipogenesis and fibrosis in settings of inflammation, aging, oxidative stress, and insulin resistance.^13^, ^14^ From this viewpoint, the combined effects of bone and muscle loss not only increase the propensity to falls and fractures but are of importance in altering the exchange of circulating factors promoting robustness versus frailty.^15^, ^16^ Clinically, increased body fat with bone and muscle loss falls under the descriptive term “sarcopenic obesity.”^17^, ^18^

The question has been raised as to whether intensive treatment of obesity might be detrimental in leading to reduction in lean body mass. Such effects have been seen with bariatric surgery^19^ and with incretin‐based treatments, described both semaglutide and with tirzepatide, although the proportional reduction in fat mass with these agents is considerably greater than that in lean body mass,^20^ leading some to argue that such approaches are unlikely to cause physical frailty or sarcopenia.^21^

Sarcopenia can be clinically assessed by measurement of handgrip strength, by using dual‐energy x‐ray absorptiometry (DXA) or bioelectrical impedance to measure muscle and fat mass, by simple history of activity tolerance, by ascertaining exercise capacity. An increasingly utilized approach to assessment of sarcopenia has been by taking advantage of two circulating body constituents used in estimating the glomerular filtration rate (GFR), creatinine, and cystatin C. Creatine synthesized in the liver is converted to phosphocreatine in muscle and brain, catalyzed by creatine kinase, with creatinine formed as a by‐product. Cystatin C is a lysosomal proteinase inhibitor produced by all nucleated cells, present in all tissues and body fluids, and like creatinine, is cleared by glomerular filtration, with eGFR‐cystatin (eGFRcys) less dependent on muscle mass than eGFR‐creatinine (eGFRcr). Given this perspective, it is reasonable to consider the creatinine/cystatin C ratio (CCR) as a measure of sarcopenia. In a metanalysis of 38 reports involving 20 362 hospitalized persons, lower CCR was associated with lower gait speed, skeletal muscle mass, and handgrip strength, and with higher mortality.^22^ Among 9894 National Health and Nutrition Examination (NHANES) 1999–2004 participants with follow‐up over a mean 15.6‐year period, lower CCR was associated with higher CV mortality.^23^ In the SPRINT (Systolic Blood Pressure Intervention Trial) trial of intensive blood pressure treatment, 502 of 2571 participants aged ≥75 years met criteria for sarcopenia using CCR and gait speed data; compared with those not having sarcopenia, this group had 1.49‐ and 1.46‐fold higher CVD event and total mortality rates, respectively.^24^ A similar analysis of 25 825 UK Biobank participants with diabetes, without diabetic microvascular complications (DMCs) at baseline, analyzed the initial eGFRcr and eGFRcys, finding those with lower eGFRcr than eGFRcys to have higher levels of inflammatory markers and BMI as well as greater likelihood to have hypertension, cigarette use, and a history of CVD; over a 13.6‐year follow‐up, the development of DMCs tracked with this measure of sarcopenia.^25^

Given this strong evidence of a relationship between sarcopenia and adverse outcome, there has been great interest in the development of measures to increase muscle regeneration, with a variety of approaches. Suggestions include various dietary approaches, including supplements with branched chain amino acids, N‐3 fatty acids, vitamin D, and agents to change the gut microbiome. As an example of the latter dietary modification, in a study of 5368 participants in NHANES, those reporting higher intake of live microorganisms such as yoghurt and kimchi had 37% lower likelihood of having sarcopenia based on DXA analysis.^26^ In addition, proposals have been made to administer hormone supplements including growth hormone, testosterone in men, and estrogen and progesterone in postmenopausal women, and pharmacologic agents including metformin, sodium‐glucose counter‐transport inhibitors, thiazolidinediones, glucagon‐like peptide 1 receptor activators, and dipeptidyl peptidase‐4 inhibitors, cell therapy approaches with myoblasts, and muscle stem cells.^12^ Exercise training has been strongly recommended to avoid sarcopenia, and there is certainly evidence that such approaches, particularly using resistance training, reduce visceral fat, increase fat‐free mass, increase strength, and reduce inflammatory cytokine levels, with reduction in triglyceride and HbA1c levels among persons with diabetes,^27^ leading to consensus statements suggesting that exercise therapy be more widely utilized.^28^ A randomized controlled 2 × 2 factorial trial of liraglutide 3.0 mg daily and/or a supervised exercise program showed that the combination of the two approaches led to greater weight loss and reduction in body fat^29^; preservation of bone mass was also greatest with combined treatment,^30^ but the effect on muscle mass has not been reported. The evidence of benefit of all of these treatment approaches among adults with frailty has, however, been questioned, with an extensive review concluding that clinical trials of a wide variety of hormonal, exercise, and nutritional approaches have shown low to very low certainty of benefit.^7^

We are left, then, with sarcopenia as a common clinical condition, affecting up to one‐sixth of the elderly, occurring somewhat more commonly among persons with diabetes, and readily ascertainable with clinical history, with measures of strength, measures of body composition, or determination that serum creatinine levels are disproportionately low in comparison to levels of cystatin C. Sarcopenia is associated with inflammation, insulin resistance, and relative or absolute increase in fat mass. The extremely potent weight loss agents semaglutide and tirzepatide may be associated with significant reduction in lean body mass, raising the question as to whether they may over time lead to an excess of sarcopenia. Crucially, persons with sarcopenia have greater likelihood of CVD and, among those with diabetes, of neuropathy, nephropathy, and retinopathy. It therefore appears logical to conclude that therapeutic approaches to restore muscle mass and strength are needed, with the caveat that such be demonstrated to lead to improvement in outcome.
